# Hemodynamic variables and progression of acute kidney injury in critically ill patients with severe sepsis: data from the prospective observational FINNAKI study

**DOI:** 10.1186/cc13161

**Published:** 2013-12-13

**Authors:** Meri Poukkanen, Erika Wilkman, Suvi T Vaara, Ville Pettilä, Kirsi-Maija Kaukonen, Anna-Maija Korhonen, Ari Uusaro, Seppo Hovilehto, Outi Inkinen, Raili Laru-Sompa, Raku Hautamäki, Anne Kuitunen, Sari Karlsson

**Affiliations:** 1 Department of Anaesthesia and Intensive Care, Lapland Central Hospital, Rovaniemi, Finland; 2 Intensive Care Unit, Division of Anaesthesia and Intensive Care Medicine, Department of Surgery, Helsinki, Finland; 3 Department of Clinical Sciences, University of Helsinki, Helsinki, Finland; 4 ANZIC-RC, Department of Epidemiology and Preventive Medicine, Monash University, Melbourne, Australia; 5 Intensive Care Unit, Kuopio University Hospital, Kuopio, Finland; 6 Department of Anaesthesia and Intensive Care Medicine, South Karelia Central Hospital, Lappeenranta, Finland; 7 Department of Anaesthesia and Intensive Care Medicine, Turku University Hospital, Turku, Finland; 8 Department of Anaesthesia and Intensive Care Medicine, Central Finland Central Hospital, Jyväskylä, Finland; 9 Department of Anaesthesia and Intensive Care Medicine, Vaasa Central Hospital, Vaasa, Finland; 10 Department of Intensive Care Medicine, Tampere University Hospital, Tampere, Finland

## Abstract

**Introduction:**

Knowledge of the association of hemodynamics with progression of septic acute kidney injury (AKI) is limited. However, some recent data suggest that mean arterial pressure (MAP) exceeding current guidelines (60–65 mmHg) may be needed to prevent AKI. We hypothesized that higher MAP during the first 24 hours in the intensive care unit (ICU), would be associated with a lower risk of progression of AKI in patients with severe sepsis.

**Methods:**

We identified 423 patients with severe sepsis and electronically recorded continuous hemodynamic data in the prospective observational FINNAKI study. The primary endpoint was progression of AKI within the first 5 days of ICU admission defined as new onset or worsening of AKI by the Kidney Disease: Improving Global Outcomes (KDIGO) criteria. We evaluated the association of hemodynamic variables with this endpoint. We included 53724 10-minute medians of MAP in the analysis. We analysed the ability of time-adjusted MAP to predict progression of AKI by receiver operating characteristic (ROC) analysis.

**Results:**

Of 423 patients, 153 (36.2%) had progression of AKI. Patients with progression of AKI had significantly lower time-adjusted MAP, 74.4 mmHg [68.3-80.8], than those without progression, 78.6 mmHg [72.9-85.4], *P* < 0.001. A cut-off value of 73 mmHg for time-adjusted MAP best predicted the progression of AKI. Chronic kidney disease, higher lactate, higher dose of furosemide, use of dobutamine and time-adjusted MAP below 73 mmHg were independent predictors of progression of AKI.

**Conclusions:**

The findings of this large prospective multicenter observational study suggest that hypotensive episodes (MAP under 73 mmHg) are associated with progression of AKI in critically ill patients with severe sepsis.

## Introduction

Both the incidence of severe sepsis and acute kidney injury (AKI) are increasing [1-3]. The incidence of AKI among patients with severe sepsis is 40 to 50% and sepsis accounts for half of the cases of AKI in the intensive care unit (ICU) [1,4-7]. Patients with septic AKI have worse outcome than septic patients without AKI in terms of longer ICU and hospital stays and higher mortality [6,7].

The understanding of the underlying pathophysiology of septic AKI is still limited [5,8]. Previously, the reduction in renal blood flow has been proposed to be essential for the establishment of AKI [9]. However, this explanation alone is inadequate. The pathogenesis of septic AKI is complex, involving apoptosis [10], inflammatory responses, and changes in microcirculation [5,8,11]. The blood flow to the organs is pressure-dependent outside the values of the autoregulatory threshold. However, a recent study reported that the autoregulation of renal blood flow is deranged in critical illness prior to and during AKI, and varies with cardiac output [12].

Current guidelines suggest norepinephrine and fluid therapy to maintain mean arterial pressure (MAP) ≥60-65 mmHg for sufficient renal perfusion and prevention of AKI in critically ill patients [11,13]. However, limited knowledge exists of the association of hemodynamics, MAP in particular, with progression of AKI during the early phase of severe sepsis [14,15]. As other options for treatment or prevention of AKI are scarce [13], better knowledge of the association of hemodynamic factors is essential. We hypothesized that higher MAP would be independently associated with a lower risk of progression of AKI.

Accordingly, in this predefined substudy of the prospective, multicenter FINNAKI study [16], we scrutinized the associations of hemodynamic variables, especially MAP, with progression of AKI in patients with severe sepsis.

## Materials and methods

The ethics committee of the Helsinki University Hospital gave approval for the study and for a deferred consent policy. Written, informed consent was obtained from the patient or patient’s proxy.

### Patients

**Figure 1:**
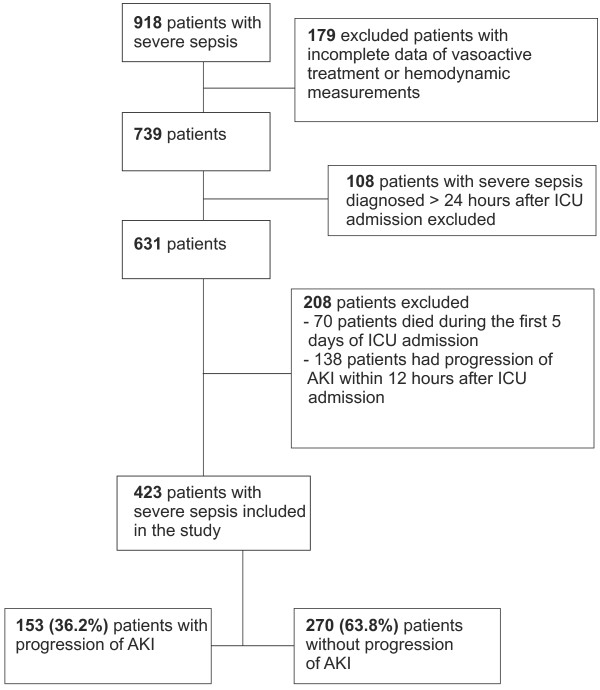
**Flowchart of study patients with severe sepsis with or without primary endpoint.** Primary endpoint = progression of acute kidney injury (AKI) = new onset of AKI (Kidney Disease: Improving Global Outcomes (KDIGO) stages 1 to 3, including initiation of renal replacement therapy) or worsening of AKI by at least one KDIGO stage during the first 5 days of ICU admission.

We identified all patients with severe sepsis and electronically recorded continuous hemodynamic data from the prospective observational FINNAKI study that was conducted in 17 Finnish ICUs between 1 September 2011 and 1 February 2012 [16]. First, we excluded four ICUs, in which median values of hemodynamic parameters were registered for periods of more than 10 minutes, or the data on vasoactive treatment were incomplete. Second, we excluded patients with severe sepsis diagnosed later than 24 h after ICU admission. Third, we excluded patients who died during the first five days in the ICU (Additional file 1 presents data on these patients) or who reached the primary endpoint within 12 h after ICU admission. The numbers of study patients and excluded patients are presented in Figure 1.

### Definitions

We defined severe sepsis according to the American College of Chest Physicians/Society of Critical Care Medicine (ACCP/SCCM) Consensus Conference Committee definition [17]. We used the Kidney Disease: Improving Global Outcomes (KDIGO) criteria to define and stage AKI according to changes in serum creatinine (SCr) and urine output [18]. According to KDIGO criteria, AKI is defined by an increase in SCr by ≥26.5 μmol/l within 48 h, or an increase in SCr ≥1.5 times baseline value, or urine output less than <0.5 ml per kg/h for six hours. We used the last SCr value from the previous year excluding the week before the ICU admission as baseline SCr, and for those without a baseline value (n = 292) we estimated it using the modification in diet in renal disease (MDRD) equation [19], assuming a glomerular filtration rate (GFR) of 75 ml per minute/1.73 m^2^. When available, we also used SCr values within 48 h before ICU admission to identify the acute increase in SCr. We defined the primary endpoint (progression of AKI) as follows: 1) new onset of AKI (KDIGO stages 1 to 3, including initiation of renal replacement therapy, RRT) or 2) worsening of AKI by at least one KDIGO stage during the first 5 days of ICU admission. The negative primary endpoint was defined as absence of AKI within the first 5 days in the ICU. Chronic kidney disease (CKD) was defined as structural or functional abnormalities of the kidney or GFR <60 ml per minute/1.73 m^2^ at least one week prior to ICU admission [20]. Hypotension within 48 h prior to ICU admission was defined as systolic blood pressure <90 mmHg for 1 h and hypovolemia according to the judgement of the physician. The attending physician set the targeted MAP level according to local practice and current sepsis guidelines [21].

### Data collection

We prospectively collected routine data (demographics, diagnosis by International Classification of Diseases (ICD-10), ICU scores, physiologic measures, and outcome) to the Finnish Intensive Care Consortium database maintained by Tieto Ltd, Helsinki, Finland [16]. Additionally, we completed a standardized case report form (CRF) at admission, and daily during days one to five in the ICU, and at ICU and hospital discharge. The CRF data comprised data on chronic and present health information, risk factors for AKI, severe sepsis, infections and antimicrobial treatment, organ dysfunction, fluid balance, and information on RRT [16]. The KDIGO stage was calculated continuously for each patient based on every measured creatinine value and hourly urine output [16]. We also prospectively collected data on hemodynamic measurements and vasopressor and inotrope treatment for this substudy.

The MAP data were collected into the database as median values of 2 or 5 minutes depending on the local patient data management system. Before collection to the database all data were manually validated for the first 24 hours of ICU admission to eradicate erroneous values. We converted all MAP data into 10-minute median values (MAP values) for all analyses.

### Data analyses

We first calculated the area under the curve (AUC) for MAP values using the NCSS 8 software (Kaysville, UT, USA) by placing the MAP values (10-minute medians) on the y-axis and time of MAP registrations as 10-minute periods on the x-axis (see Additional file 2: Figures S1A, B, and C). We adjusted the MAP AUCs with the total aggregate time of MAP registrations (that is, the sum of 10-minute periods of MAP median values) for each patient during the first 24 h (= time-adjusted MAP). For patients who reached the endpoint within 24 h, MAP registrations were included in the analysis until the endpoint was reached. The point of time of the highest AKI stage was the time of reaching the endpoint. For patients who did not reach the endpoint within 24 h, MAP values of the first 24 h were included in the analysis.

Second, we calculated the MAP AUC under threshold values of MAP: 55, 60, 65, 70, 75, 80, 85 mmHg as the area of MAP and aggregate time of MAP values beneath each threshold. Third, we calculated the aggregate time and adjusted aggregate time (percentage) of MAP below threshold values (55, 60, 65, 70, 75, 80, 85 mmHg) for each patient. Fourth, we calculated the time-adjusted MAP deficit below threshold values (55, 60, 65, 70, 75, 80, 85 mmHg) by dividing the MAP AUC below each threshold value with the total aggregate time of MAP values for each patient (Additional file 2: Figures S1A, B, and C). Fifth, we identified patients with time-adjusted MAP below the best cutoff value for prediction of AKI progression based on the receiver operating characteristic (ROC) analysis of the time-adjusted MAP. Sixth, the time-adjusted MAP below this level was used as a categorical variable in the multivariable regression analysis.

We identified the highest blood lactate value, the lowest blood pH value and the lowest base excess (BE) values for each patient for the first 24 h and during days one to five. The worst values of the first 24 h were used in the analysis. The highest dose of norepinephrine, epinephrine, dopamine, dobutamine, and vasopressin for the first 24 h were used in the analyses. We calculated the vasopressor load using the following formula: vasopressor load (μg/kg/minute) = norepinephrine (μg/kg/minute) + dopamine (μg/kg/minute/2) + epinephrine (μg/kg/minute) + phenylephrine (μg/kg/minute/10) [22,23]. In the study ICUs, phenylephrine infusions were not used, and thus, it was not included in the vasopressor load. We defined treatment with dobutamine, milrinone or levosimendan as inotrope treatment, epinephrine was analyzed as a vasopressor only [24]. We calculated the time-adjusted fluid balance for the first day in ICU by dividing the total fluid balance by the number of hours in the ICU at fluid balance registration. We defined hydroxyethyl starch (HES) and gelatine as colloids.

### Statistical analyses

We present the data as absolute number (percentage) or median with IQR. For continuous data, we used the Mann-Whitney *U*-test for comparison of groups. For categorical data, we used the chi-square test or Fisher’s exact test, when appropriate. We analyzed the association of hemodynamic data and risk factors for progression of AKI with the primary endpoint by univariable analysis. We then included prognostic factors with *P* <0.2 into a multivariable forward conditional regression analysis to test the possible independent association with the primary endpoint. We analyzed the ability of time-adjusted MAP and highest dose of norepinephrine to predict worsening of AKI by calculating the AUC by ROC analysis with the primary endpoint. We assessed the best cutoff value by the Youden Index (sensitivity + specificity -1) [25]. We performed all statistical analyses using IBM SPSS Statistics 19.0 and 20.0 (IBM, Armonk, NY, USA) or NCSS 8 (Kaysville, UT, USA) software.

## Results

### Incidence of AKI and progression of AKI

We included 423 patients with severe sepsis in the study (Figure 1). Of these 423 patients, 153 (36.2%) had AKI and presented with primary endpoint (progression of AKI) within 5 days of ICU admission. Patients with progression of AKI more often suffered from septic shock (134/153, 87.6%) compared to those without progression (185/270, 68.5%), *P* <0.001. They also had CKD, diabetes mellitus, suffered from hypovolemia and hypotension prior to ICU admission and had received radiocontrast dye preceding ICU admission more often than those without progression of AKI (Table 1). The 90-day mortality of patients with severe sepsis with progression of AKI was higher than for patients with severe sepsis without progression of AKI (32.7% versus 18.9%, *P* = 0.001). The ICU mortality did not differ significantly between the groups (7.8% versus 3.3% respectively, *P* = 0.06).

**Table 1:** Characteristics of patients with severe sepsis with or without progression of AKI

	**Data available**	**Progression of AKI**	**Data available**	**No progression of AKI**	***P*-value**
		**N = 153**		**N = 270**	
Age, years	153	64.0 (51.0 to 78.0)	270	63.0 (52.0 to 73.0)	0.1
Gender, male	153	92 (60.1)	270	184 (68.1)	0.1
Baseline creatinine available	153	108 (70.6)	270	184 (68.1)	0.6
Comorbidity					
Hypertension	153	81 (52.9)	270	126 (46.7)	0.22
Systolic heart failure or arteriosclerosis	153	30 (19.6)	270	45 (16.7)	0.45
COPD	153	15 (9.8)	270	38 (14.1)	0.2
Chronic kidney disease	153	17 (11.1)	270	7 (2.6)	<0.001
Chronic liver disease	153	8 (5.2)	270	12 (4.4)	0.72
Diabetes mellitus	153	42 (27.5)	270	50 (18.5)	0.032
Hypotension prior to ICU	151	64 (42.4)	267	75 (28.1)	0.003
Hypovolemia prior to ICU	151	78 (51.7)	268	96 (35.8)	0.002
Radiocontrast dye prior to ICU	153	44 (28.8)	268	52 (19.4)	0.03
Emergency admission	153	148 (96.7)	270	267 (98.9)	0.12
Operative admission	153	49 (32.0)	270	59 (21.9)	0.02
Community acquired infection	152	76 (50.0)	270	137 (50.7)	0.88
Source of infection					
Pulmonary	139	70 (50.4)	246	155 (63.0)	0.02
Abdominal	139	45 (32.4)	246	52 (21.1)	0.02
Genitourinary	139	14 (10.1)	246	12 (4.9)	0.05
Soft tissue	139	14 (10.1)	246	28 (12.4)	0.7
SAPS II points	153	43.0 (35.0 to 55.0)	270	38.0 (30.0 to 46.0)	<0.001
SAPS II points without age and renal components	153	24.0 (18.0 to 30.0)	270	24.0 (17.0 to 31.0)	0.7
SOFA D1 points	153	9.0 (7.0 to 11.0)	270	7.0 (5.0 to 9.0)	<0.001
SOFA D1 points, without renal points	153	8.0 (6.0 to 10.0)	270	7.0 (5.0 to 9.0)	0.001
During ICU stay					
Mechanical ventilation	153	117 (76.5)	270	168 (62.2)	0.003
Use of sepsis corticosteroids	151	55 (36.4)	264	46 (17.4)	<0.001
Any vasoactive treatment	153	134 (87.6)	270	181 (67.0)	<0.001
Furosemide	153	131 (85.6)	270	189 (70.0)	<0.001
Dose of furosemide (iv) per ICU day, mg/day	153	13.6 (3.5-33.9)	270	4.2 (0.0-16.0)	<0.001
Aminoglycoside antibiotics	153	1 (0.7)	270	6 (2.2)	0.22
Peptidoglycan antibiotics	153	16 (10.5)	270	29 (10.7)	0.93
ACE inhibitor or ARB	153	10 (6.5)	270	26 (9.6)	0.27
NSAID	153	5 (3.3)	270	17 (6.3)	0.18
Radiocontrast dye	153	15 (9.8)	270	33 (12.2)	0.45
Length of stay					
ICU	153	5.7 (3.3 to 10.3)	270	3.8 (2.0 to 7.0)	<0.001
Hospital	153	16.0 (9.5 to 26.5)	270	15.0 (9.0 to 23.8)	034
90-day mortality	153	50 (32.7)	270	51 (18.9)	0.001

Values are expressed as median (IQR) or count (percentage). Progression of acute kidney injury (AKI) is defined as onset of new AKI or worsening of AKI by at least one Kidney Disease: Improving Global Outcomes (KDIGO) stage within the first 5 days after ICU admission. COPD, chronic obstructive pulmonary disease; chronic kidney disease (CKD) was defined as structural or functional abnormalities of the kidney or glomerular filtration rate (GFR) <60 ml/minute/1.73 m^2^ at least one week prior to ICU admission; hypotension, systolic blood pressure <90 mmHg for 1 h; hypovolemia, by judgement of clinicians; SAPS II, simplified acute physiology score, SOFA D1, sequential organ failure assessment, first score during the ICU stay; iv, intravenous; ACE, angiotensin converting enzyme; ARB, angiotensin II receptor blocker; NSAID, non-steroidal anti-inflammatory drug.

Of these 423 patients, 102 (24.1%) had new onset of AKI and 51 (12.1%) had worsening of AKI by at least one KDIGO stage. The highest AKI stage was based on changes in SCr in 80.1% (339/423), urine output 13.9% (59/423) and by initiation of RRT in 25 cases (5.9%). The progression of AKI is illustrated in Additional file 3. The median time for reaching the endpoint was 27.0 hours (16.5 to 45.5 hours). Of 153 patients with progression of AKI, 66 (43.1%) reached the endpoint on the admission day, and 50 patients (32.7%) on the second day in the ICU. RRT was initiated in 34/423 (8%) of the study patients.

### MAP and progression of AKI

**Figure 2:**
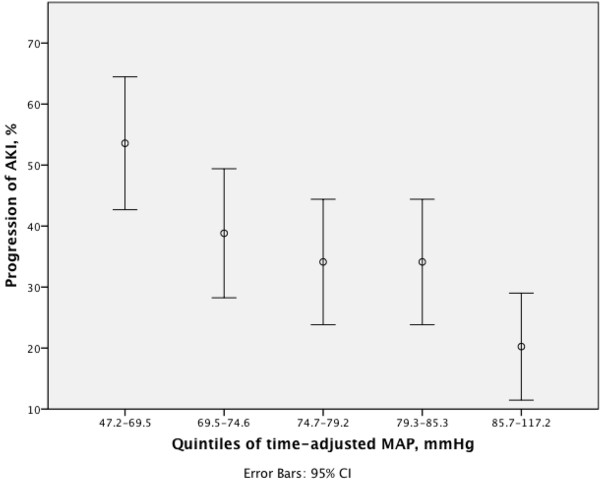
**Progression of acute kidney injury (AKI) by quintiles of time-adjusted mean arterial pressure (MAP).** The incidence of progression of AKI divided in quintiles of time-adjusted MAP presented for patients with severe sepsis during the first 24 h in the ICU.

We included 53,724 10-minute medians of MAP values in the calculations. The median aggregate MAP registration time for patients who fulfilled the endpoint was 1,230 (945 to 1,430) minutes compared to 1,420 (1,350 to 1,440) minutes in those who did not fulfill the endpoint. Patients with progression of AKI had significantly lower time-adjusted MAP, 74.4 mmHg (68.3 to 80.8), than those without progression, 78.6 mmHg (72.9 to 85.4), *P* <0.001 (Additional file 4). The time-adjusted MAP and aggregate times of MAP values below MAP thresholds of patients with or without progression of AKI are presented in Table 2. Except for threshold level 85 mmHg (*P* = 0.07), the MAP AUC below thresholds (55 to 80 mmHg) were larger in patients with progression of AKI than without (*P* >0.05 for all) (Additional file 5). The time-adjusted MAP deficits were larger for all threshold levels (55 mmHg and 85 mmHg, *P* <0.05 for all) (Additional file 5). The Youden index of the time-adjusted MAP yielded a cutoff value of 72.7 mmHg for best prediction of AKI progression, (ROC AUC 0.63; CI 95% 0.58 to 0.69), sensitivity 0.44; CI 95% 0.36 to 0.52), specificity 0.76; CI 95% 0.71 to 0.81). The incidence of AKI progression divided by quintiles of time-adjusted MAP is shown in Figure 2.

**Table 2:** Time-adjusted mean arterial pressure (MAP) and vasoactive treatments divided by progression of acute kidney injury

	**Progression of AKI**	**No progression of AKI**	***P*-value**
	**N = 153**	**N = 270**	
**Time-adjusted MAP**	74.4 (68.3 to 80.8)	78.6 (72.9 to 85.4)	<0.001
**Time-adjusted MAP below 73 mmHg (%)**	69 (45.1)	68 (25.2)	<0.001
**Aggregate time below MAP thresholds, minutes**			
55 mmHg	0.0 (0.0 to 10.0)	0.0 (0.0 to 10.0)	0.02
60 mmHg	10.0 (0.0 to 70.0)	5.0 (0.0 to 30.0)	0.007
65 mmHg	80.0 (10.0 to 280.0)	50.0 (0.0 to 160.0)	0.02
70 mmHg	290.0 (80.0 to 620.0)	180.0 (40.0 to 480.0)	0.02
75 mmHg	600.0 (235.0 to 985.0)	490.0 (160.0 to 870.0)	0.15
80 mmHg	770.0 (445.0 to 1140.0)	750.0 (287.5 to 1102.5)	0.35
85 mmHg	910.0 (660.0 to 1260.0)	1015.0 (567.5 to 1270.0)	0.86
**Time adjusted aggregate time below MAP thresholds, %**			
55 mmHg	0.0 (0.0 to 1.1)	0.0 (0.0 to 0.7)	0.01
60 mmHg	1.1 (0.0 to 7.2)	0.4 (0.0 to 2.5)	0.002
65 mmHg	7.7 (0.8 to 27.3)	3.6 (0.0 to 11.2)	0.002
70 mmHg	25.4 (7.0 to 59.5)	14.8 (3.4 to 34.6)	<0.001
75 mmHg	56.9 (23.0 to 81.9)	37.3 (12.2 to 65.3)	<0.001
80 mmHg	76.3 (43.5 to 95.0)	56.5 (24.7 to 80.7)	<0.001
85 mmHg	93.1 (65.5 to 97.9)	75.8 (45.8 to 92.8)	<0.001
**Norepinephrine,** n (%)	**131 (85.6)**	**180 (66.7**)	>0.001
Max dose ≤24 h in ICU (μg/kg/minute)	0.19 (0.07 to 0.42)	0.08 (0.00 to 0.19)	<0.001
Max dose 1 to 5 d in ICU (μg/kg/minute)	0.24 (0.11 to 0.50)	0.14 (0.08 to 0.30)	<0.001
**Epinephrine,** n (%)	**5 (3.3)**	**2 (0.7)**	0.1
Max dose ≤24 h in ICU (μg/kg/minute)	0.02 (0.02 to 0.02)	0.14 (0.02 to 0.66)	0.31
Max dose 1 to 5 d in ICU (μg/kg/minute)	0.15 (0.02 to 0.15)	0.14 (0.06 to 0.78)	0.8
**Dopamine,** n (%)	**2 (1.3)**	**2 (0.7)**	0.62
Max dose ≤24 h in ICU (μg/kg/minute)	5.0 (2.7 to 5.0)	9.1 (5.56 to 9.1)	0.13
Max dose 1 to 5 d in ICU (μg/kg/minute)	5.0 (2.7 to 5.0)	9.1 (5.56 to 9.1)	0.13
**Vasopressor load max **(μg/kg/minute)			
≤24 h in ICU	0.32 (0.15 to 0.85)	0.14 (0.08 to 0.29)	<0.001
1 to 5 d in ICU	0.40 (0.22 to 0.96)	0.15 (0.08 to 0.30)	<0.001
**Vasopressin, **n (%)	4 (2.6)	1 (0.4)	0.06
**Dobutamine,** n (%)	33 (21.6)	15 (5.6)	<0.001
**Levosimendan,** n (%)	11 (7.2)	4 (1.5)	<0.002
**Milrinone**, n (%)	4 (2.6)	1 (0.4)	0.06

Values are expressed as count (percentage) or median (IQR). The median doses of drug doses are calculated for number of patients receiving aforementioned drug. AKI, acute kidney injury; AUC, area under the curve; Vasopressor load max (μg/kg/minute) = norepinephrine max (μg/kg/minute) + dopamine max (μg/kg/minute/2) + epinephrine max (μg/kg/minute).

### Vasopressor and inotrope treatment

Of the 423 patients with severe sepsis, 311 (73.5%) were treated with norepinephrine within the first 5 days of ICU admission, and 293 (69.3%) patients received norepinephrine during the first 24 h in ICU. Patients with progression of AKI received norepinephrine more often (*P* <0.001) during days 1 to 5 in the ICU. The maximum dose of norepinephrine was higher both during the first 24 h (*P* <0.001) and within the first 5 days in the ICU (*P* <0.001). Patients with progression of AKI also received inotropes more often than patients with no AKI progression, 26.1% versus 7.4%, *P* <0.001. Table 2 presents details of vasopressor and inotrope treatment.

**Figure 3:**
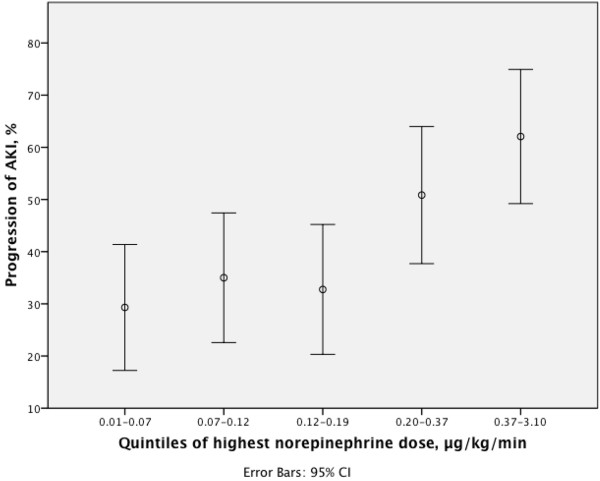
**Progression of acute kidney injury (AKI) by quintiles of highest dose of norepinephrine.** The incidence of progression of AKI divided in quintiles of highest dose of norepinephrine is presented for patients with severe sepsis during the first 24 h in the ICU.

Patients with progression of AKI by quintiles of highest norepinephrine dose are presented in Figure 3. When patients were divided into quintiles according to time-adjusted MAP, the highest dose of norepinephrine during 24 h was significantly associated with AKI progression in the lowest quintile of time-adjusted MAP (47.2 to 69.5 mmHg) (*P* <0.001), but not in the four higher quintiles (*P* = 0.33, *P* = 0.92, *P* = 0.16 and *P* = 0.78 respectively). The maximum vasopressor load was higher in patients with progression of AKI than in patients without progression of AKI (*P* <0.001).

The Youden index of the highest norepinephrine dose yielded a cutoff value of 0.19 μg/kg/minute mmHg for best prediction of AKI progression (ROC AUC 0.66, CI 95% 0.60 to 0.71; sensitivity 0.44, CI 95% 0.37 to 0.52; specificity 0.80, CI 95% 0.75 to 0.85).

Patients with time-adjusted MAP below 73 mmHg and highest dose of norepinephrine over 0.19 μg/kg/minute (42/61 patients, 68.9%) developed AKI more frequently than patients with time-adjusted MAP over 73 mmHg and norepinephrine below 0.19 μg/kg/minute (58/226 patients, 25.7%), odds ratio (OR) 6.40, 95% CI 3.45 to 11.89.

### Other factors associated with progression of AKI

Of the 423 patients, 68 (16.1%) received HES and 89 (21.0%) received gelatine within 48 h preceding ICU admission. Within the first 5 days of ICU admission patients with progression of AKI received colloids (type not specified) more often than those without (74.5% versus 59.3%, *P* = 0.002). The fluid balance on admission day was significantly higher in patients with progression of AKI (112.8 ml/h, 7.4 to 216.4 ml/h) than among those without progression, 51.7 ml/h, -20.5 to 138.6 ml/h), *P* <0.001). Patients with progression of AKI had higher blood lactate levels, lower blood pH levels and lower BE values than patients without progression both during the first 24 h and during days 1 to 5 (*P* <0.001 for all) (Additional file 6). Hypertension as co-morbidity prior to ICU admission was not associated with progression of AKI (*P* = 0.23). Time-adjusted MAP was higher in patients with hypertension than in patients without hypertension; 78.5 mmHg (72.7 mmHg to 84.2 mmHg) versus 75.6 (70.3 mmHg to 82.9 mmHg) respectively, *P* = 0.04).

### Multivariable logistic regression analysis

Results of the univariable and multivariable regression analyses are shown in Table 3.

**Table 3:** Univariable and multivariable regression analyses for factors associated with progression of AKI in patients with severe sepsis

	**Univariable analysis**		**Multivariable analysis**			
			**Model 1**		**Model 2**	
	**Odds ratio (95% CI)**	***P*-value**	**Odds ratio (95% CI)**	***P*-value**	**Odds ratio (95% CI)**	***P*-value**
CKD	4.696 (1.901, 11.600)	0.001	6.72 (2.19, 20.63)	0.001	7.24 (2.36, 22.23)	0.001
Diabetes mellitus	1.665 (1.041, 2.662)	0.033	NS		NS	
Hypotension prior to ICU	1.883 (1.239, 2.863)	0.003	NS		NS	
Radiocontrast dye prior to ICU	1.677 (1.056, 2.664)	0.029	NS		NS	
Operative admission	1.685 (1.079, 2.631)	0.022	NS		NS	
Abdominal infection	1.786 (1.117, 2.855)	0.015	NS		NS	
SOFA D1 without renal point	1.136 (1.056, 1.223)	0.001	NS		NS	
Use of sepsis corticosteroids	2.715 (1.715, 4.298)	<0.001	NS		NS	
Use of dobutamine within first 24 h in the ICU	4.607 (2.259, 9.395)	<0.001	2.42 (1.00, 5.81)	0.049	2.68 (1.11, 6.48)	0.028
Norepinephrine max dose 24 h	4.234 (2.036, 8.803)	<0.001	NS		NS	
Daily dose of furosemide (iv, mg)	1.006 (1.002, 1.009)	0.001	1.00 (1.00, 1.01)	0.002	1.01 (1.00, 1.01)	0.001
Fluid balance per hour on D1 in ICU	1.002 (1.000, 1.003)	0.005	NS		NS	
Lactate 24 h highest	1.374 (1.218, 1.549)	<0.001	1.36 (1.18, 1.57)	<0.001	1.35 (1.17, 1.55)	<0.001
Time-adjusted MAP	0.952 (0.931, 0.973)	<0.001	0.96 (0.94, 0.99)	0.006	-	
Time-adjusted MAP below 73 mmHg	2.440 (1.602, 3.716)	<0.001	-		2.57 (1.48, 4.46)	0.001

CKD, chronic kidney disease defined as structural or functional abnormalities of the kidney or glomerular filtration rate (GFR) <60 ml/miute/1.73 m^2^ at least one week prior to ICU admission; hypotension, systolic blood pressure <90 mmHg for 1 h; SOFA D1, sequential organ failure assessment, first score during the ICU stay; iv, intravenous; MAP, mean arterial pressure; NS, not significant.

No significant interaction between time-adjusted MAP and dose of norepinephrine was detected.

First, to the first regression model the time-adjusted MAP was entered to describe MAP. The highest lactate value during the first 24 h, CKD, daily dose of intravenous furosemide, and time-adjusted MAP per mmHg (OR 0.96, 95% CI 0.94 to 0.99) remained independent predictors of progression of AKI.

Second, instead of MAP as a continuous covariate, we tested time-adjusted MAP below the cutoff value of 73 mmHg as a categorical variable. The highest lactate value during the first 24 h, CKD, daily dose of intravenous furosemide per mg, use of dobutamine during the first 24 h, and time-adjusted MAP below 73 mmHg (OR 2.57, 95% CI 1.48 to 4.46) remained independent predictors of progression of AKI.

## Discussion

In this large prospective multicenter observational study, progression of AKI occurred in 36% of patients with severe sepsis. CKD, lactate level, dose of intravenous furosemide, use of dobutamine, and lower time-adjusted MAP or time-adjusted MAP below 73 mmHg were independently associated with progression of AKI.

Few studies have assessed the relationship of hemodynamics and progression of AKI during early phases of severe sepsis [14,15]. Maintaining mean arterial pressure over 60 to 65 mmHg is currently suggested to maintain adequate renal blood flow and perfusion [11,13]. However, the true value of MAP that is beneficial for the kidney is unknown.

In the present study we found that patients with progression of AKI had significantly lower time-adjusted MAP (74 mmHg), than those without progression (79 mmHg). The best cutoff MAP level for prediction of AKI progression was 73 mmHg. Our finding is in line with recent studies, which have demonstrated that higher MAP levels than previously recommended may be required to maintain adequate renal perfusion [14,15]. An experimental study showed similar results in septic pigs [26]. In the subgroup analysis of 127 patients with septic shock Badin *et al*. found that patients who developed AKI had significantly lower time-averaged MAP than those who did not. The authors concluded that MAP between 72 and 82 mm Hg could be necessary to prevent AKI in patients with septic shock [14]. In another recent retrospective cohort study of 274 septic patients, blood pressure was associated with need for RRT, maximal creatinine concentrations, and urine output. MAP under 75 mmHg predicted the need for RRT. Consequently, the authors suggested that for renal protection, a MAP level of at least 75 mmHg may be beneficial [15].

There is evidence that lower MAP is associated with worse outcome in patients with septic shock with AKI [15,27]. Recently, a small study of cardiac surgery patients showed that renal oxygen delivery and GFR improved when MAP was restored from 60 mmHg to 75 mmHg [28]. Current literature suggests that inflammatory processes and changes in renal microcirculation, with subsequent uncoupling of systemic and renal blood flow, may be central processes in the pathophysiology of AKI [5,8,11]. Renal autoregulation is disturbed during critical illness and its dependence on cardiac output is increased [12]. Thus, maintaining adequate renal perfusion to overcome derangements caused by loss of autoregulation may be one of the few current therapeutic options for prevention and treatment of AKI.

In the present study, when patients were divided by level of time-adjusted MAP into quintiles, the highest dose of norepinephrine was independently associated with progression of AKI in patients in the lowest quintile of time-adjusted MAP (47.2 to 69.5 mmHg). Our data showed that in the lowest quintile, the incidence of AKI progression increased with increasing doses of norepinephrine. These results may suggest that progression of AKI is more likely when higher doses of norepinephrine are required to maintain targeted blood pressure levels in more severely ill patients with severe sepsis, plausibly by causing excess constriction of regional vascular beds [29]. It may also reflect the impact of more severe illness and more profound vascular hyporesponsiveness on the progression of AKI [30].

Norepinephrine is the vasopressor of choice recommended for restoration of MAP in acute circulatory failure during sepsis [21]. Even though norepinephrine may have deleterious effects on renal blood flow and renal function in healthy subjects [31,32], it may increase renal perfusion and GFR in patients with circulatory failure [28,33]. However, in an experimental study norepinephrine failed to increase renal microcirculation in septic pigs, in spite of improved perfusion pressure [34]. There is also evidence of the association of adverse outcome in septic shock with increasing vasopressor load [23]. As renal blood flow and perfusion may show individual variation, the evaluation of renal blood flow, and its distribution and resistive index has been proposed for finding the optimal MAP target for each patient [33,35]. With better knowledge of the individual optimal MAP excessive use of vasopressors may be avoided.

Higher blood lactate, lower pH and lower BE were associated with progression of AKI indicating, that systemic hypoperfusion was present, even though cardiac function was not included in this study. Hence, the use of inotropes may be explained by attempts to increase insufficient cardiac output in patients with progression of AKI. However, low BE and pH may also be consequences of AKI. Recent data indicate that dobutamine may not improve microcirculatory perfusion in septic shock despite an increase in cardiac index [36]. In addition, some inotropes may have independent deleterious effects on the septic kidney [37]. In agreement with prospective randomized studies [38,39] indicating that the use of colloids in the ICU cause AKI, we also found an association between colloids and progression of AKI.

This study has some limitations. First, although patient data were collected prospectively, patients were not randomized to treatment arms targeting different MAP values or vasopressors, inotropes or fluid treatment. Thus, the association of time-adjusted MAP, and time-adjusted MAP deficits below threshold values, as well as higher vasopressor and fluid load to progression of AKI may partly be explained by the impact of more severe illness. Second, exclusion of patients who died during the first five days may have caused selection bias by elimination of the most severely ill patients (Additional file 1). However, inclusion of patients who died would also cause bias. Progression of AKI and death may be considered as competing risk, as patients may die before progression of AKI is identified. Nevertheless, some patients could have died without progression of AKI. Third, this study focused on MAP rather than mean perfusion pressure of the kidney, as intra-abdominal pressure (IAP) was measured only in a few patients, and information on IAP levels in the majority of patients was lacking. Fourth, data on cardiac function or mixed venous oxygen saturation were collected in only a minority of patients monitored with a pulmonary artery catheter. Therefore, the associations between low cardiac output or low mixed venous oxygen saturation during the early phase of severe sepsis and progression of AKI could not be assessed in this study. Finally, during the FINNAKI study we only collected data on the type of colloids received during 48 h prior to ICU admission. Therefore, we could not assess the association of the use of colloids, nor on the use of different colloids, particular HES, with progression of AKI.

## Conclusions

In this large prospective study of patients with severe sepsis, we found that time-adjusted MAP was significantly lower and independently associated with progression of AKI in these patients. Our findings suggest that avoiding hypotensive episodes (MAP under 73 mmHg) may prevent progression of AKI. This hypothesis should be confirmed in a prospective randomized trial.

## Key messages

• Time-adjusted MAP under 73 mmHg was associated with progression of AKI in critically ill patients with severe sepsis

• The highest dose of norepinephrine was not associated with progression of AKI except in patients in the lowest quintile (47.2 to 69.5 mmHg) of time-adjusted MAP

## Abbreviations

ACCP/SCCM: American College of Chest Physicians/Society of Critical Care Medicine; AKI: Acute kidney injury; AUC: Area under curve; BE: Base excess; CKD: Chronic kidney disease; CRF: Case report form; DIC: Disseminated intravascular coagulation; GFR: Glomerular filtration rate; HES: Hydroxyethyl starches; IAP: Intra abdominal pressure; ICD-10: International Classification of Diseases; KDIGO: Kidney disease: improving global outcomes; MAP: Mean arterial pressure; MDRD: Modification of diet in renal disease; OR: Odds ratio; ROC: Receiver operating characteristic; RRT: Renal replacement therapy; SAPS II: Simplified acute physiology score; SCr: Serum creatinine; SOFA: Sequential organ failure assessment.

## Competing interests

The authors declare to have no competing interests.

## Authors’ contributions

MP and EW performed the data analysis and drafted the manuscript (equal contribution). STV and VP participated in designing the study and critically revised the manuscript. AMK, AU, KMK, SH, OI, RLS, RH, AK and SK critically revised the manuscript. All authors participated in the data collection and read and approved the final manuscript.

## Supplementary Material

Additional file 1: Table S1 Characteristics of patients who died within the first 5 days in the ICU. Click here for file

Additional file 2: Figure S1 A, B, and C. Examples of registered mean arterial pressures (MAP) and MAP area under curve (AUC) during the first 24 h in the ICU. Click here for file

Additional file 3: Figure S2 Onset and progression of acute kidney injury (AKI) from the first stage of AKI to the highest stage of AKI during the first 5 days in the ICU. Click here for file

Additional file 4: Figure S3 Time-adjusted mean arterial pressure stratified by progression of acute kidney injury (AKI). Click here for file

Additional file 5: Table S2 Area under curve of mean arterial pressure (MAP AUC) under threshold values and time-adjusted MAP deficit below MAP thresholds divided by progression of acute kidney injury (AKI). Click here for file

Additional file 6: Table S3 Acid-base balance and plasma lactate values between patients with or without progression of acute kidney injury (AKI). Click here for file
