# Supplementary material for: Hemodynamic variables and progression of acute kidney injury in critically ill patients with severe sepsis: data from the prospective observational FINNAKI study
Source: Crit Care. 2013 Dec 13;17(6):R295. doi: 10.1186/cc13161 (PMC4056430; doi:10.1186/cc13161)
Supplement: Additional file 1: Table S1 — Characteristics of patients who died within the first 5 days in the ICU. [file cc13161-S1.docx]

**Additional file 1 A Table S1.**

Characteristics of patients who died within the first five days in the ICU

Values are expressed as median (IQR) or count (percentage).

SAPS II Simplified Acute Physiology Score, SOFA D1 Sequential Organ Failure Assessment, first score during the ICU stay; AKI acute kidney injury; KDIGO Kidney Disease: Improving Global Outcomes; RRT renal replacement therapy; ICU intensive care unit

|  | Death within first 5 days in the ICU  N= 70 |
| --- | --- |
| Age (years) | 70.5 (58.5-80.0) |
| Gender (male) | 38 (54.3) |
| Emergency admission | 70 (100) |
| Operative admission | 10 (14.3) |
| SAPS II points | 67.50 (58.8-78.3) |
| SOFA D1 points | 13.0 (10.0-15.0) |
| SOFA D1 renal points | 2.5 (0.8-4.0) |
| Any AKI | 53 (75.7) |
| KDIGO Stage 1 | 12 (17.1) |
| KDIGO Stage 2 | 10 (14.3) |
| KDIGO Stage 3 | 31 (44.3) |
| Time to the first AKI (hours) | 7.0 (2.0-14.3) |
| Time to the highest AKI (hours) | 12.0 (4.3-18.8) |
| Time to RRT (hours) | 13.0 (5.5-32.0) |
| Septic shock | 69 (98.6) |
| Time to death from ICU admission (hours) | 16.6 (9.1-50.1) |
