# Supplementary material for: Hemodynamic variables and progression of acute kidney injury in critically ill patients with severe sepsis: data from the prospective observational FINNAKI study
Source: Crit Care. 2013 Dec 13;17(6):R295. doi: 10.1186/cc13161 (PMC4056430; doi:10.1186/cc13161)
Supplement: Additional file 2: Figure S1 — A, B, and C. Examples of registered mean arterial pressures (MAP) and MAP area under curve (AUC) during the first 24 h in the ICU. [file cc13161-S2.pdf]

Additional file 1. Figure S 1 A, B and C.

1. MAP AUC was calculated using NCSS 8 as area of MAP (mmHg [y-axis)]) x time (minutes [x-axis])). Figure A shows the MAP AUC of patient 510077.

A.

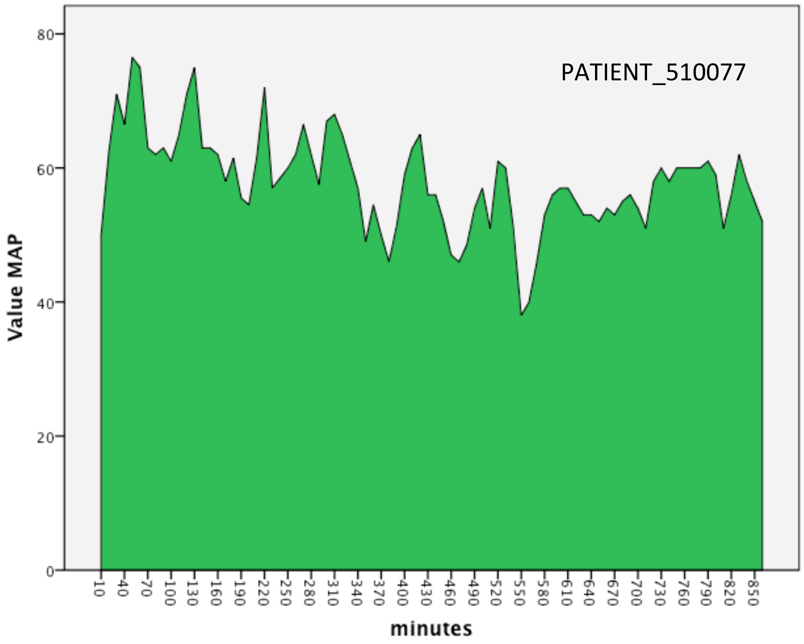

2. MAP AUC under thresholds for each patient: area of MAP x time under thresholds for each patient (Figure B. The arrows show MAP AUC under 55 mg for patient 510077)

B.

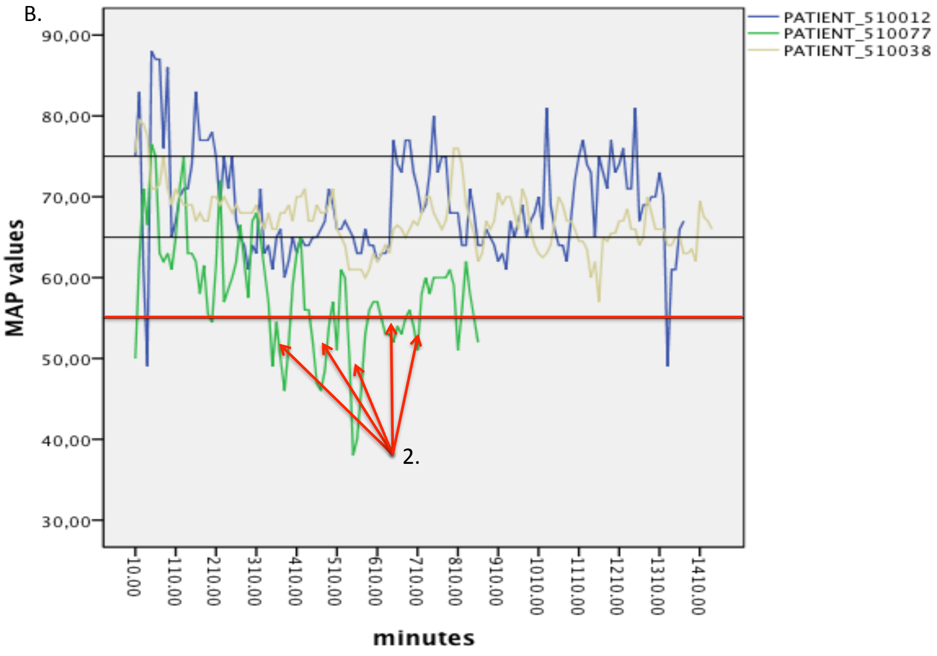

3. Time-adjustment was performed by dividing the MAP AUC (paragraph.1) with the actual aggregate time of MAP measurements. MAP AUC / time in minutes for each patient (x-axis) = time-adjusted MAP: Patient 510038: 95972.5mmHgmin / 1440 min=66.6 mmHg, Patient 510077 49335.0 mmHgmin/ 860 min = 57.4 mmHg

C.

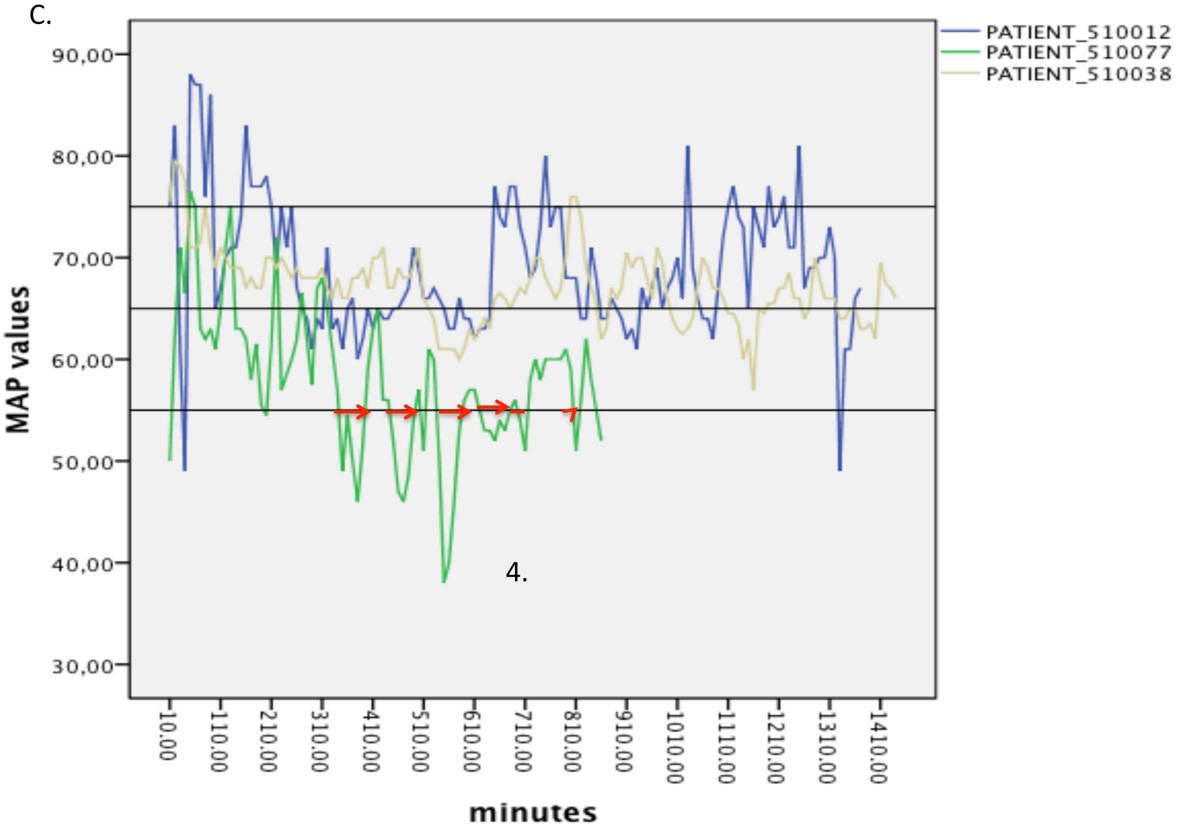

4. Aggregate time of MAP below MAP thresholds (55-85 mmHg) was calculated as the total number of MAP values (10-minute median) below each MAP threshold value x 10 minutes. (Depicted by the arrows in Figure C.)
5. Adjusted aggregate time below MAP was calculated as aggregate time of MAP below threshold divided by total aggregate time of MAP measurements. Patient 510077: 270 min below 55 mmHg / 860 min =31.4 %
6. Time-adjusted MAP deficit below thresholds was calculated as MAP AUC beneath thresholds divided by the total aggregate time of MAP measurements. See paragraphs 2 and 5. For patient 510077: 1300 mmHgmin / 860 min= 1.5 mmHg at threshold 55 mmHg.
