# Supplementary figures and images for: Hemodynamic variables and progression of acute kidney injury in critically ill patients with severe sepsis: data from the prospective observational FINNAKI study
Source: Crit Care. 2013 Dec 13;17(6):R295. doi: 10.1186/cc13161 (PMC4056430; doi:10.1186/cc13161)

# FIRST AKI

# HIGHEST AKI

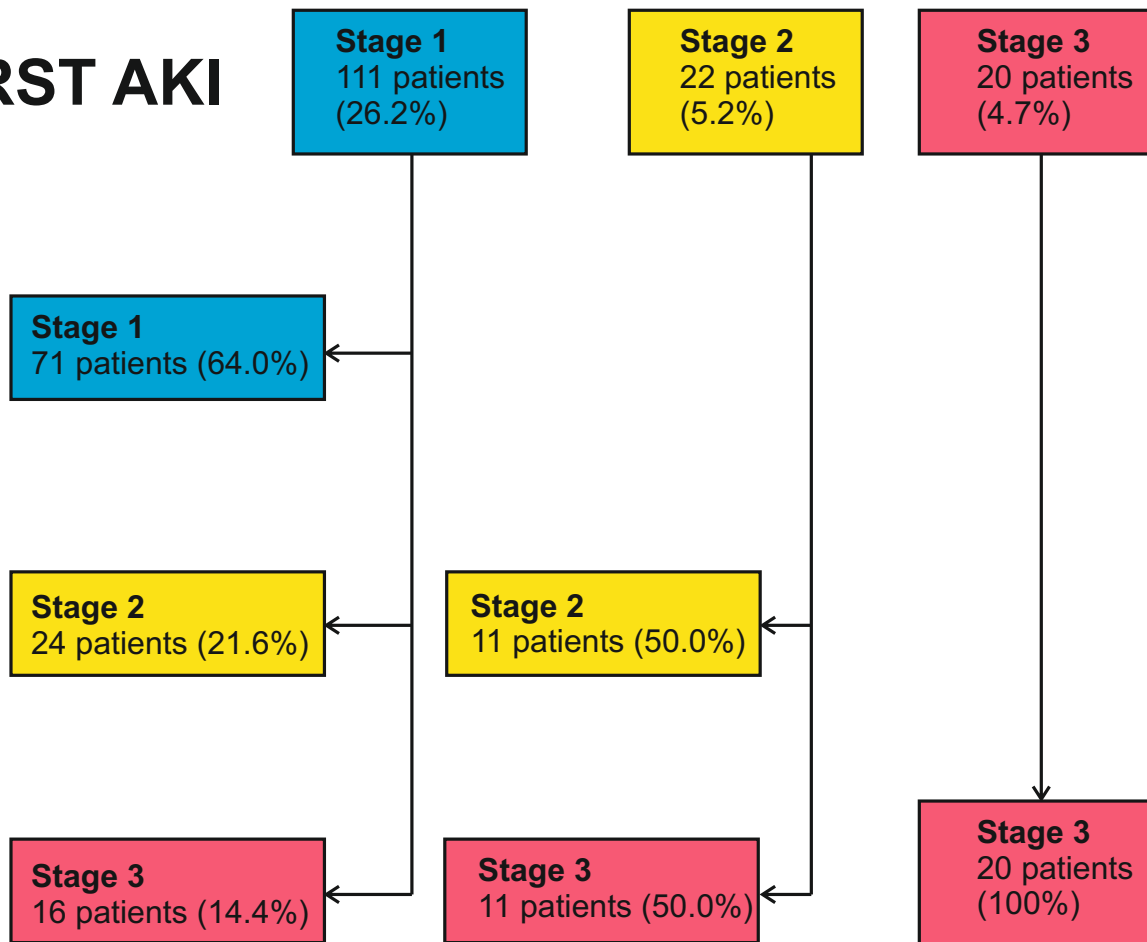

Supplement: Additional file 3: Figure S2 — Onset and progression of acute kidney injury (AKI) from the first stage of AKI to the highest stage of AKI during the first 5 days in the ICU. [file cc13161-S3.pdf]

Time-adjusted MAP, mmHg

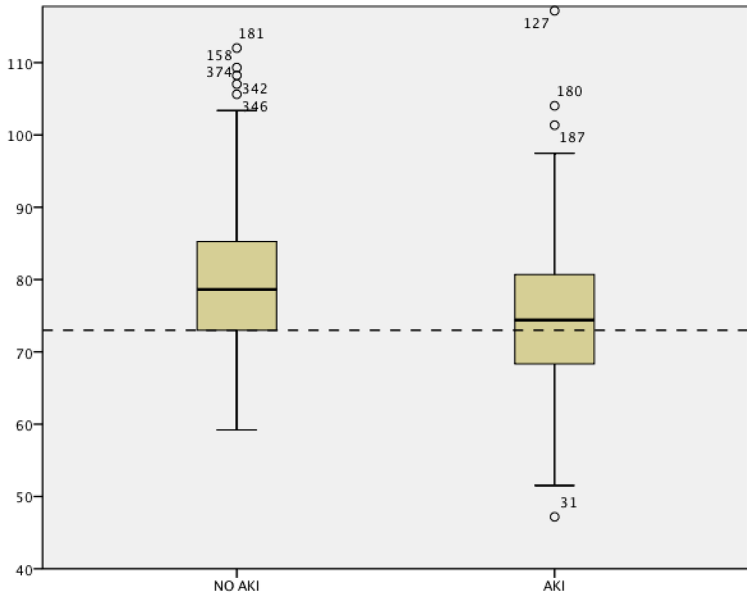

Supplement: Additional file 4: Figure S3 — Time-adjusted mean arterial pressure stratified by progression of acute kidney injury (AKI). [file cc13161-S4.pdf]
