# Supplementary material for: Hemodynamic variables and progression of acute kidney injury in critically ill patients with severe sepsis: data from the prospective observational FINNAKI study
Source: Crit Care. 2013 Dec 13;17(6):R295. doi: 10.1186/cc13161 (PMC4056430; doi:10.1186/cc13161)
Supplement: Additional file 5: Table S2 — Area under curve of mean arterial pressure (MAP AUC) under threshold values and time-adjusted MAP deficit below MAP thresholds divided by progression of acute kidney injury (AKI). [file cc13161-S5.docx]

**Additional file 5. Table S2**. Area under curve of mean arterial pressure (MAP AUC) under threshold values and time-adjusted MAP deficit below MAP thresholds divided by progression of AKI.

| MAP thresholds | No progression of AKI N=270 | Progression of AKI  N=153 | p-value |
| --- | --- | --- | --- |
| **MAP AUC below thresholds (mmHgmin)** | | | |
| 55 mmHg | 0.0 (0.0-0.0) | 0.0 (0.0-35.0) | 0.03 |
| 60 mmHg | 2.5 (0.0-121.3) | 30.0.0 (0.0-222.5) | 0.01 |
| 65 mmHg | 152.5 (0.0-613.8) | 265.0 (20.0-1095.0) | 0.04 |
| 70 mmHg | 797.5 (157.5-2218.8.) | 1190.0 (232.5-3400.0) | 0.02 |
| 75 mmHg | 2407.5 (641.3-5537.5) | 3925.0 (1097.5-7415.0) | 0.02 |
| 80 mmHg | 5742.5 (1775.0-10611.3) | 7325.0 (2592.5-12232.5) | 0.03 |
| 85 mmHg | 10005.0 (3911.3-16603.8) | 11825.0 (5522.5-18257.5) | 0.07 |
| **Time-adjusted MAP deficit below thresholds (mmHg)** | | | |
| 55 mmHg | 0.00 (0.00-0.00) | 0.00 (0.00-0.03) | 0.02 |
| 60 mmHg | 0.00 (0.00-0.09) | 0.03 (0.00-0.23) | 0.005 |
| 65 mmHg | 0.13 (0.00-0.47) | 0.24 (0.02-1.02) | 0.007 |
| 70 mmHg | 0.60 (0.13-1.69) | 1.18 (0.22-3.14) | 0.001 |
| 75 mmHg | 1.97 (0.51-4.31) | 3.13 (1.01-7.05) | <0.001 |
| 80 mmHg | 4.32 (1.43-7.95) | 7.02 (2.81-11.31) | <0.001 |
| 85 mmHg | 7.71 (3.37-12.01) | 16.12 (6.02-16.12) | <0.001 |
