# Supplementary material for: Hemodynamic variables and progression of acute kidney injury in critically ill patients with severe sepsis: data from the prospective observational FINNAKI study
Source: Crit Care. 2013 Dec 13;17(6):R295. doi: 10.1186/cc13161 (PMC4056430; doi:10.1186/cc13161)
Supplement: Additional file 6: Table S3 — Acid-base balance and plasma lactate values between patients with or without progression of acute kidney injury (AKI). [file cc13161-S6.docx]

**Additional file 6. Table S3.** Acid-base balance and plasma lactate values between patients with or without progression of AKI. Values are the worst values within the first 24 hours and the worst values prior to endpoint for patients with progression of AKI and for those without progression the worst value within the first five days in the intensive care unit. Values are expressed as median (IQR). AKI Acute kidney injury, ICU Intensive care unit, BE base excess

|  | Data  available | Progression of AKI (N=153) | Data  available | No progression of AKI (N=270) | P-value |
| --- | --- | --- | --- | --- | --- |
| **Values ≤24hrs in the ICU** | | | | | |
| pH (minimum) | 146 | 7.32 (7.22-7.36) | 230 | 7.37 (7.31-7.41) | <0.001 |
| BE (minimum) | 146 | -5.2 (-9.8- (-2.4)) | 230 | -1.8 (-4.3-0.6) | <0.001 |
| Lactate max mmol/l | 136 | 3.1 (2.0-4.8) | 219 | 2.0 (1.5-2.9) | <0.001 |
| **Values** | **prior to endpoint** | | **during the first five ICU days** | | |
| pH (minimum) | 146 | 7.31(7.22-7.36) | 230 | 7.35 (7.30-7.40) | <0.001 |
| BE (minimum) | 146 | -5.7 (-9.9- (2.7)) | 231 | -2.0 (-4.3- 0.1) | <0.001 |
| Lactate mmol/l | 135 | 2.9 (1.8-4.6) | 219 | 1.9 (1.4-2.9) | <0.001 |
